# Recurrent “outsider” intronic variation in the *SLC5A*6 gene causes severe mixed axonal and demyelinating neuropathy, cyclic vomiting and optic atrophy in 3 families from Maghreb

**DOI:** 10.3389/fgene.2024.1352006

**Published:** 2024-01-29

**Authors:** Lamisse Mansour-Hendili, Cyril Gitiaux, Madeleine Harion, Céline Latouche, Bénédicte Heron, Tanya Stojkovic, Mélanie Rama, Thomas Smol, Anne Sophie Jourdain, Karine Mention, Yann Nadjar, Manuel Schiff, Julie Lemale, Jamal Ghoumid, Frédéric Gottrand, Cécile Talbotec, Agnès Rötig, Benoît Funalot, Isabelle Desguerre

**Affiliations:** ^1^ Département de Biochimie-Biologie Moléculaire, Pharmacologie, Génétique Médicale, AP-HP, Hôpitaux Universitaires Henri Mondor, Créteil, France; ^2^ IMRB Equipe Pirenne, Laboratoire d’excellence LABEX GRex, Université Paris-Est Créteil, Créteil, France; ^3^ Université Paris Cité, Paris, France; ^4^ Service de Neurophysiologie Clinique Pédiatrique, Centre de Référence des Pathologies Neuromusculaires, Hôpital Necker-Enfants Malades, Assistance Publique Hôpitaux de Paris, Paris, France; ^5^ Université de Médecine, Sorbonne Université, Paris, France; ^6^ INSERM, Paris, France; ^7^ Service de Neuropédiatrie, APHP, Hôpital Trousseau, Paris, France; ^8^ Centre de Référence des maladies Neuromusculaires Nord/Est/Ile-de-France Institut de Myologie, Hôpital Pitié-Salpêtrière, Paris, France; ^9^ Institut de Génétique, CHU Lille, Lille, France; ^10^ University of Lille, ULR7364-RADEME, Lille, France; ^11^ Service de Biochimie et Biologie Moléculaire, CHU Lille, Lille, France; ^12^ Centre de Référence des Maladies Héréditaires du Métabolisme, Service Néphrologie, Endocrinologie, Maladies Métaboliques et Hématologie Pédiatrique, Hôpital Jeanne de Flandre, Lille, France; ^13^ Département de Neurologie UF Neuro-Métabolisme, Centre de Référence des maladies Métaboliques et Lysosomales à expression Neurologique (CRML-Neuro), APHP-SU, Hôpital Pitié-Salpêtrière, Paris, France; ^14^ Reference Centre for Mitochondrial Disorders (CARAMMEL), Reference Centre for Inherited Metabolic Diseases (MaMea), Hôpital Necker-Enfants-Malades, APHP, Paris, France; ^15^ Imagine Institute, Genetics of Mitochondrial Disorders, INSERM, Paris, France; ^16^ Department of Paediatric Nutrition and Gastroenterology, Reference Centre for Rare Digestive Diseases, Trousseau Hospital, APHP, Paris, France; ^17^ CHU Lille, Clinique de Génétique, Guy Fontaine, Lille, France; ^18^ University Lille, CHU Lille, Infinite U1286 Inserm, Lille, France; ^19^ Service de gastroentérologie, hépatologie et Nutrition Pédiatrique, Hôpital Jeanne de Flandre, CHU Lille, Lille, France; ^20^ Pediatric Gastroenterology-Hepatology-Nutrition, Hôpital Necker-Enfants Malades, Paris, France; ^21^ Université Paris-Est Créteil, INSERM, IMRB, Créteil, France; ^22^ Paediatric Neurology Department, Necker-Enfants malades University Hospital, Assistance Publique Hôpitaux de Paris, Hôpital Necker-Enfants Malades, Paris, France

**Keywords:** multivitamin deficiency, intronic variation, splicing, recurrent, mixed neuropathy, optic atrophy

## Abstract

Sodium dependent multivitamin transporter (SMVT) deficiency is a very rare autosomal recessive disorder characterized by multisystemic clinical manifestations due to combined biotin, panthotenic acid and lipoic acid deficiency. About 10 families have been described so far. Accurate diagnosis is crucial because of the possibility of a supplementation treatment with proven efficacy. Here we describe 4 new patients (3 additional families) originating from the same world region (Algeria, Maghreb). All patients, born form consanguineous parents, were homozygous carriers of the same intronic variation, outside of canonical sites, in the *SLC5A6* gene encoding SMVT. RNA study in one family allowed confirming the pathogenic effect of the variation and re-classifying this variant of uncertain significance as pathogenic, opening the possibility of genetic counseling and treatment. The identification of the same variation in three distinct and apparently unrelated families is suggestive of a founder effect. The phenotype of all patients was very similar, with systematic optic atrophy (initially considered as a very rare sign), severe cyclic vomiting, and rapidly progressive mixed axonal and demyelinating sensory motor neuropathy.

## Introduction

Human Sodium dependent multivitamin transporter (hSMVT) deficiency is a very rare genetic disorder, which has been reported so far in only 17 patients from 10 families (Subramanian et al., 2017; Byrne et al., 2019; Schwantje et al., 2019; Hauth et al., 2022; Holling et al., 2022; Montomoli et al., 2023; Rupasinghe and Onyeador, 2023; Utsuno et al., 2023). SMVT is a Na + dependent vitamin transporter encoded by the *SLC5A6* gene. SMVT transports biotin (vitamin B7) and pantothenic acid (vitamin B5) which are water soluble vitamins and the lipoic acid (Quick and Shi, 2015). Its expression is highest in intestinal epithelia and brain. In humans, Biotin and panthotenic acid are crucial for development and are only provided by alimentation (Neophytou and Pitsouli, 2022). Biallelic pathogenic variations of the *SLC5A6* gene cause hSMVT deficiency, an autosomal recessive disorder first described by Subramanian et al., in 2017 (Subramanian et al., 2017). The phenotypic spectrum of the disease, comprising variable multisystemic manifestations, has been considerably extended since the first report. The first reported patients showed failure to thrive, developmental delay or early normal development followed by developmental regression, seizures, diarrhea or vomiting, immunodeficiency, and/or osteopenia (Subramanian et al., 2017; Byrne et al., 2019; Schwantje et al., 2019). Then Holling *et al.* expanded the phenotypic spectrum by reporting five individuals from three families with mixed sensory motor axonal and demyelinating neuropathies associated with optic atrophy, recurrent infections, and repeated episodes of chronic intestinal pseudo-obstruction (Holling et al., 2022). Byrne *et al* described progressive mixed sensory motor polyneuropathy in 2019 in one patient. Very recently, Utsuno *et al.* described a new phenotypical entity due to SMVT deficiency and called it SRDDBC for “spontaneously remitting developmental delay with brain cysts” due to missense variants having a milder effect on the function of the transporter. They propose in their study a classification of SMVT deficiency in three categories: SMVT deficiency (SMVTD, ubiquitous spatial localization of phenotype and very early onset), SRDDBC which is a milder form and childhood-onset biotin-responsive peripheral motor neuropathy (COMNB) (Holling et al., 2022).

We report here 4 individuals from 3 distinct families originating from Algeria with the same homozygous intronic variation in the SLC5A6 gene and very similar manifestations in all patients, associating mixed severe and progressive sensory motor axonal and demyelinating neuropathy, optic atrophy and severe cyclic vomiting.

### Material and methods

#### Sequencing and data analysis

Whole exome sequencing (WES) has been performed for all four patients described in this study (Cf Supplementary Material for details concerning method and bioinformatics analysis).

#### RNA study

RNA study has been performed in patient 3 on blood and fibroblasts samples (Cf Supplementary Material for details concerning method).

#### Clinical data

All clinical data are summarized in Table 1.

**TABLE 1:** Clinical findings.

	Patient 1	Patient 2	Patient 3	Patient 4
Consanguinity	yes	yes	yes	yes
Age at first symptom	4 Y	4 Y	4 Y	3 Y
Mild Cognitive delay	5 Y	5 Y	no	no
Pyramidal signs	5 Y	4 Y	6 Y	no
Optic atrophy	6 Y	6 Y	6 Y	10 Y
Neuropathy	8 Y mixed	8 Y mixed	5 Y mixed	7 Y mixed
Ambulation	with support at 14 Y	loss at 10 Y	loss at 5 Y	with support
				at 17 Y
Biochemical analysis*	N	N	N	Abnormal
				Elevated 3-OH-valeric acid
First line genetic analyses**	N	N	N	N
Cardiac	Wolf Parkinson	no	no	no
Brain and medullar MRI including MRSI	normal except OA	OA, WM occipital Ab	cortical atrophy	normal except OA
Lower limb contractures	severe	severe	severe	severe distal
Epilepsy	no	no	no	no
Growth delay	no	8 Y	5 Y	8 Y/11 Y
Cyclic vomiting	8 Y	6 Y	4 Y	12 Y
CIPO	no	7 Y	5 Y	12 Y
Parenteral nutrition	no	13 Y (3 m)	5 Y	13 Y
Enteral nutrition	no	13 Y (6 m)	5 Y	13 Y
Bladder palsy	no	no	5 Y	recurrent acute at 13 Y
High blood pressure	no	no	no	13 Y
Medical treatment	amitriptylin	amitriptylin	amitriptylin	amitriptylin + oxcarbazepin; gabapentin
Vitamines uptake	no	no	low doses	beflavine at 13Y B5/B7/lipoic acid high doses at 22 Y
			carnitine	
Long term follow up	15 Y	14 Y	death at 7 Y	23 Y

^a^ Biochemical analysis (patient 1, 2, 3, 4): redox points, OAC, AAC, homocystein; VLCFA, phytanic, pristanic, pipecolic acids, plasma bile acid intermediates, acylcarnitin profil, lysosomal enzymes activity, thymidine phosphorylase activity, transferrine electrophoresis; CSF, analysis: normal (patient 2,3); Muscle biopsy (patient 2, 3, 4): no clear respiratory chain deficit. No ragged red fibers.

^b^ First line Genetic analysis (patient 1, 2, 3, 4): ACPA, Mt DNA (including search for deletion, *MERRF, MELAS, NARP*, genes), *ECGF1*/MNGIE, gene; NGS, mitochondrial genes (including *POLG1, POLG2, RRM2B*), NGS, neuropathy; NGS, optic atrophy (*MFN2, OPA1, OPA2, GJB1, TMEM, 126A, OPA7*), PDH, exploration and E2/lipoylation sub-units (*NFU1, BOLA3, IBA57, LIAS, LIPT1*).

Abbreviations in Table 1: OA, optic atrophy; WM, white matter; Y, years; CSF, cerebrospinal fluid; MRI, magnetic resonance imaging; MRSI, magnetic resonance spectroscopic imaging; CIPO, Chronic intestinal pseudo-obstruction; AAC, amino acids chromatography; OAC, organic acids chromatography; ACPA, micro array DNA; Mt DNA, mitochondrial DNA; NGS, next-generation sequencing; VLCFA, very-long-chain fatty acids; NCS/EMG, (nerve conduction study and electromyography); MNGIE, mitochondrial neurogastrointestinal encephalomyopathy; PDH, pyruvate dehydrogenase; SMVT, sodium dependent multivitamin transporter.

Patient 1 is a 15 years old male. He was born from consanguineous parents and had 2 brothers among which one was also affected (patient 2). He was able to walk at 11 months of age. A Wolf Parkinson White syndrome was diagnosed and treated at 3 years. Progressive walking disorder associating peripheral neuropathy and spastic diplegia began at 4 years. In the same time, optic atrophy revealed by decrease of vision with nystagmus was present and a language delay was noticed. Cyclic vomiting (approximatively 4 days every 2 months with complete food intolerance needing fluid supplementation) began at 6 years of age associated with recurrent fever conducting to recurrent hospitalizations in emergency for IV rehydration. He needed orthopedic surgery for severe contractures of the lower limbs. Large metabolic investigations during the acute digestive episods were normal. NCS/EMG (nerve conduction study and electromyography) confirmed the axonal sensory motor neuropathy (Table 2). Cerebral MRI (except optic atrophy) was normal. Genetic investigations (micro-array DNA, Mt DNA and NGS analysis for nuclear mitochondrial gene) were normal. At age 14, he was able to walk with support. Recurrent vomiting were partly responsive to amitriptyline chlorhydrate.

**TABLE 2:** Electromyography results.

Patient			Patient 2		Patient 3						Patient 4			
Age (years)			8		4		5		7		15		23	
Motor nerve conduction		Normal value	R	L	R	L	R	L	R	L	R	L	R	L
DML (ms)	Median	< 4.2	NR	3.10	3.60	3.33	4.91	6.41	A	NR	8.4	5.9	A	A
	Ulnar	< 3.5	NR	2.90	4.5	NR	4.30	3.46	A	NR	3.1	5.9	3.7	3.8
	Common Peroneal	< 5.0	NR	4.91	NR	NR	NR	NR	NR	NR	5.2	A	A	A
	Tibial	<5.5	6.88	5.38	4.37	6.01	NR	NR	A	NR	5.5	6.3	A	A
	Median	> 5	NR	5.0	3.5	4.7	0.29	0.18	A	NR	0.24	0.77	A	A
CMAP (mV)	Ulnar	>5	NR	4.1	2.5	NR	0.24	1.63	A	NR	5.48	6.19	1.53	1.08
	Common Peroneal	>3	NR	0.61	NR	NR	NR	NR	NR	NR	0.32	A	A	A
	Tibial	>3	0.85	1.02	0.05	0.19	NR	NR	A	NR	0.17	0.06	A	A
	Median	> 50	NR	39.5	30.7	34.1	18.1	NR	A	NR	NR	34	A	A
MNCV (m/s)	Ulnar	> 50	NR	55.4	32.6	NR	NR	43.7	A	NR	41	41	37	39
	Common Peroneal	> 42	NR	32.9	NR	NR	NR	NR	NR	NR	41	40	A	A
	Tibial	> 42	36.8	31.6	28.9	NR	NR	NR	A	NR	NR	NR	A	A
**Sensory nerve conduction**		Normal value	R	L	R	L	R	L	R	L	R	L	R	L
SNCV (m/s)	Median (transcarpal)	> 45	NR	39.5	39.8	39.3	32.5	NR	A	NR	A	34	A	A
	Ulnar	> 45	NR	NR	NR	NR	NR	NR	NR	NR	36	35	A	A
	Radial	> 45	NR	NR	NR	NR	NR	NR	NR	NR	34	30	36	30
	Sural	> 40	A	A	33.8	NR	NR	NR	A	NR	A	A	A	A
	Superficial peroneal	>40	NR	NR	NR	NR	NR	NR	NR	NR	35	38	30	38
	Median (transcarpal)	> 15	NR	2.5	12.0	18.7	6.2	NR	A	NR	A	2	A	A
SNAP (µV)	Ulnar	> 8	NR	NR	NR	NR	NR	NR	NR	NR	6.1	0.7	A	A
	Radial	> 10	NR	NR	NR	NR	NR	NR	NR	NR	3.6	0.6	2.3	1.9
	Sural	> 10	A	A	10.1	NR	NR	NR	A	NR	A	A	A	A
	Superficial peroneal	>10	NR	NR	NR	NR	NR	NR	NR	NR	2	6.7	2.1	2.1

SNCV, sensory nerves conduction velocities; MNCV, motor nerve conduction velocities; SNAP, sensory nerves action potential; CMAP, compound muscle action potentials; DML, distal motor latency; latency; R, right; L, left; NR, not recorded; A, Absent; ENMG, results showed an sensory and motor axonal neuropathy or a mixed demyelinating and axonal neuropathy worsening over time.

Patient 2: a 14 years old male, brother of patient 1 presented the same history. He was able to walk at 12 months of age. A language delay was later noticed. At 4 years spastic diplegia with abolition of the tendon reflexes was diagnosed. Optic atrophy was diagnosed at 6 years old. Recurrent vomiting began at 6 years with weight flathering leading to undernutrition. NCS/EMG evidenced peripheral neuropathy at age 8 (Table 2). Progressive weakness resulted in loss of ambulation at age 10. Because of severe distal contractures of lower limb, he needed orthopedic surgery. Enteral nutrition and parenteral nutrition were necessary at 13 years during 6 months. Large metabolic investigations including thymidine phosphorylase activity and search for mitochondrial dysfunction were normal. Digestive explorations including enteroscan, gastric and esophageal endoscopy with biopsies were non-contributive. Muscle biopsy showed no mitochondrial dysfunction. After a long time of hospitalization because of total digestive intolerance, he was stabilized with vitamins supplementation biotin (20 mg/day), pantothenic acid (555 mg/day) and lipoic acid (300 mg/day) and amitriptyline at age 14, at 6 months of follow-up. Digestive investigations comprising oesophageal endoscopy with biopsies, rectal biopies, enteroscan, oesophagal and anorectal manometry, oeso-gastroduodenal transit, small intestin and colorectal radiography were normal.

Patient 3: this girl born of consanguineous parents experienced walking problems at 4 years of age. At age 4, she developed episodic urinary retention. Spinal cord MRI was normal and NCS/EMG (nerve conduction study and electromyography) showed mixed axonal and demyelinating sensory motor neuropathy (Table 2). Polyradiculitis was suspected at CSF analysis (proteinorachia at 0.38 g/L N: 0.15–0.6 g/L) without any autoantibodies. Large and repeated biochemical investigations were unremarkable (mitochondrial, lysosomal, acylcarnitine profile). She had pyramidal signs with severe lower limbs retractions and loss ambulation at age 5. Muscle biopsy showed a neurogenic muscular atrophy and a normal respiratory chain analysis. At 6 years she developed cyclic vomiting and multiple episodes of chronic intestinal pseudo-obstruction (CIPO). Digestive investigations comprising oesophageal endoscopy with biopsies, rectal biopies, enteroscan, oesophagal and anorectal manometry, oeso-gastroduodenal transit, small intestin and colorectal radiography were normal.

She needed nutritional support with gastrostomy and jejunostomy and at least total parenteral nutrition since few months. She died at 7 years of age after suffering quadriplegia, brainsteam dysfunction and pan digestive palsy.

Patient 4, a 23-year-old male man, was born of consanguineous parents, His oldest brother died at the age of 4 years. His two sisters were asymptomatic. At 1 year of age, he had a surgical cure of right pyelo-ureteral junction syndrome. A progressive symmetrical distal weakness of the lower limbs started at the age of 3 years, leading to clumsiness and running difficulties. At age 7 he had distal hypoesthesia, weak and amyotrophic muscles in the lower limbs, generalized osteotendinous areflexia and an axonal sensory and motor neuropathy was diagnosed. At age 8, failure to thrive was noticed, worsening at 11. At age 10, hand weakness appeared; he suffered from neuropathic pain and paresthesia treated by amitriptyline and oxcarbazepine then gabapentin; bilateral optic atrophy was diagnosed in a context of decreased visual acuity. At age 12.5, he suffered from recurrent episodes of vomiting and CIPO requiring parenteral nutrition for 2 months, associated with ileostomy, and followed by enteral nutrition through gastrostomy from age 13 to 18: oesophageal endoscopy showed Mallory Weiss syndrome during a CIPO access, proximal small intestin ileus and dyskinesia of the third lower oesophagus. Multiple biopsies, anorectal manometry, oeso-gastroduodenal transit were normal.

At age 13, he had episodic acute bladder dysfunction, and arterial hypertension treated by amlodipine and perindopril for 6 months, followed by amlodipine alone. At 17 years, bilateral cavovarus feet required a corrective surgery, after what he lost autonomous walking. NCS showed very low CMAP in lower limbs and median nerves, sensory potentials were markedly decreased in all four limbs, whereas conduction velocities were mildly diminished, overall consistent with a sensory and motor axonal neuropathy (Table 2). Brain MRI was normal, except for atrophic nerve atrophy.

Muscular biopsy showed neurogenic changes, some COX-negative muscular fibers but no specific respiratory chain complex deficit. No mitochondrial DNA pathogenic variation or deletion was identified.

Thymidine phosphorylase activity and TYMP gene sequencing were normal, as well as PDH activity and sequencing of sub-unit E2/lipoylation genes (NFU1, BOLA3, IBA57, LIAS, LIPT1). Optic atrophy panel detected no pathogenic variation in the MFN2, OPA1, OPA2, GJB1, OPA7 genes.

Other biochemical studies including transferrin electroporesis, Redox points, amino-acids and organic acids chromatographies, plasmatic homocysteine and peroxysomal dosages (VLCFA, phytanic, pristanic, pipecolic acids and plasma bile acid intermediates) were normal. But urinary measurement of 3 hydroxy valeric acid before treatment was abnormal with elevated levels: 115 μmol/mmol of creatinine (N < 18) as commonly showed in biotin deficiencies. At 23 years, he has a severe motor weakness and amyotrophic thenar muscles and wasting of lower limbs resulting in drop-foot gait. He used a wheelchair at the age of 20. He has been treated for a few months with biotin (20 mg/day), pantothenic acid (555 mg/day) and lipoic acid (300 mg/day).

#### Genetic results

**FIGURE 1:**
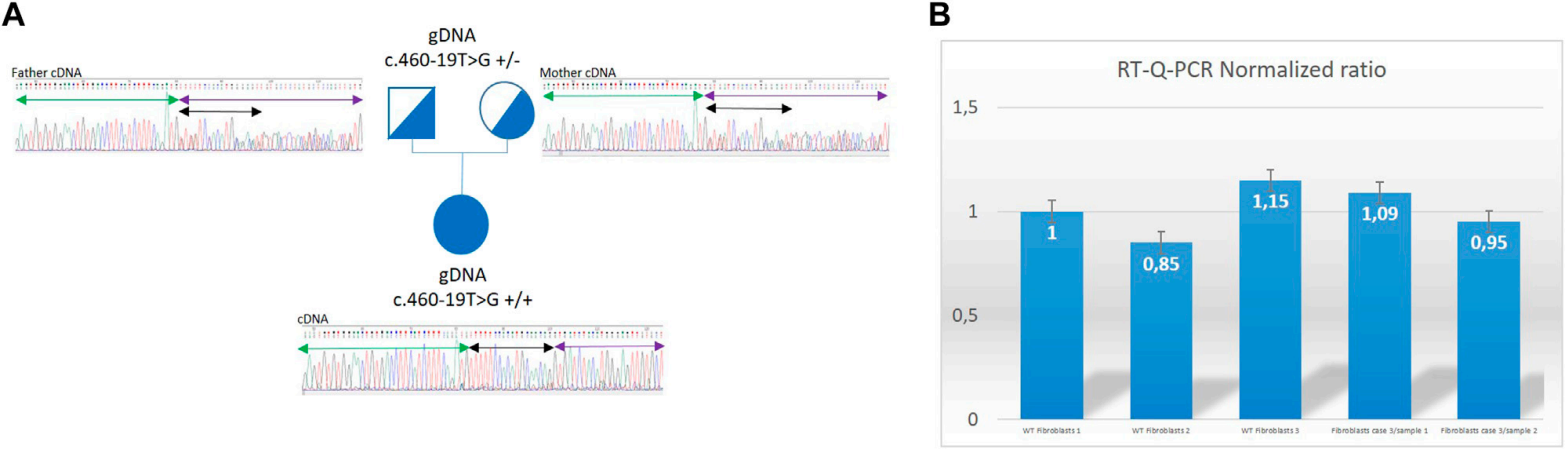
Genetic results for Patient 3. **(A)**: Family 3 pedigree showing that Sanger sequencing electrophoregrams from amplified cDNA from exon 3 to exon 6 of the *SLC5A6* gene. Mother (heterozygous for the c.460–19T>G on genomic DNA) presents on cDNA from Paxgene^®^ tube the insertion of 18 bp at heterozygous state between exon 4 and exon 5 of the gene corresponding to the last 18 nucleotides of intron and use of the cryptic acceptor site. Father (heterozygous for the c.460–19T>G on genomic DNA) presents on cDNA from Paxgene^®^ tube the insertion of 18 bp at heterozygous state between exon 4 and exon 5 of the gene corresponding to the last 18 nucleotides of intron and use of the cryptic acceptor site. Proband (homozygous for the c.460–19T>G on genomic DNA) presents on cDNA sequencing from Paxgene^®^ tube the insertion of 18 bp at homozygous state between exon 4 and exon 5 of the gene corresponding to the last 18 nucleotides of intron and use of the cryptic acceptor site. Black double arrow represents the TTTTTCCACATGCAACAG 18 bp inserted sequence. Green arrow = exon 4; purple arrow = exon 5. **(B)**: Histogram with ratios obtained in RT-qPCR Roche™ LC480 method. Expression ratios calculated for the patient 3 fibroblasts are normal showing no modification of gene expression in poband. Error bars have been estimated on the triplicate values of crossing threshold (Ct) points made for each sample. WT, wild type; WT fibroblasts 1 are used in the method of calculation of ratios as internal reference gDNA, genomic DNA.

NGS sequencing identified in all patients the same variation: c.460–19T>G (2: 27206553 (GRCh38)) at homozygous state in the *SLC5A6* gene (NM_021095). This variation was not recorded in gnomAD database v3 nor in Clinvar before our submission (our Clinvar accession number is VCV002507024.2 VCV002507024.2 - ClinVar - NCBI (nih.gov)). In silico predicting splicing tools: SpliceAI, MaxEntscan and splice site finder concluded to a probable pathogenic effect of this variation on splicing by creating a new splicing acceptor site in intron 4 sequence (Splice AI AG score 0.80). RNA study was performed on RNA extracted from Paxgene^®^ (BD-Biosciences) blood tubes for patient 3 and her parents and from patient 3’s fibroblasts. It showed the retention of 18 nucleotides in the sequence of the cDNA at homozygous state in the proband and at heterozygous state in both parents, due to the use of the new cryptic acceptor-splicing site created by the variation (Figure 1). Ratio of relative expression of the *SLC5A6* gene compared to the housekeeping gene ABL obtained in RT-q-PCR performed on the proband fibroblasts cDNA and on fibroblasts reference samples cDNA showed normal values and are not suggestive of changes in *SLC5A6* mRNA expression levels. This suggests that the translation of the abnormal *SLC5A6* mRNA results in the incorporation of 6 new amino acids (AA) in frame within the fourth transmembrane domain of the transporter (Holling et al., 2022), between AA 153 and 154.

Our study, associating family genetic and clinical data with functional mRNA tests allows to classify this variation as pathogenic according to ACMG guidelines (; on behalf of the ACMG Laboratory Quality Assurance Committee et al., 2015), with the following criteria: PP1-S, PM2, PP3, PS3.

## Discussion

We reported clinical, electro-neuromyographic and genetic data from 4 patients with the same variation in the *SLC5A6* gene, encoding human sodium dependent multivitamin transporter (hSMVT). The clinical features encompass severe cycling vomiting, sensory motor axonal or mixed neuropathy and early optic atrophy, starting in infancy and leading to severe disability. CIPO occurred in 3 out of 4 patients. First line investigations (brain MRI, biochemical tests and targeted genetic studies) did not disclose the cause of the syndromic neuropathy in these patients. The main first hypothesis for this complex disease was MNGIE (Mitochondrial Neuro GastroIntestinal Encephalomyopathy), ruled out since no demyelinating features has been seen on nerve conduction studies, and brain MRI was normal except for optic atrophy. Before genetic testing results, involvement of mitochondrial genes such as the *SLC25A46* gene or the *OPA3* gene has been evoked because of their known association with severe form of sensory motor axonal neuropathy, optic atrophy and gastrointestinal dysmobility (Abrams et al., 2015; Horga et al., 2019). But in these diseases, brain MRI usually shows white matter signal hyper-intensities which is not the case in our patients.

Thanks to WES sequencing and communication between the different teams involved in patients’ care, a final diagnosis was obtained and supplementation treatment introduced in patient 4. Nine months after the instauration of supplementation treatment in this patient, last clinical follow up did not show any worsening elements in the clinical status of the patient. Phenotype seems to be stabilized. In the previously published studies efficacy of supplementation in this disease seems to be correlated with age at beginning. Subramanian et al. began treatment at 19 months in their patient and showed motor and verbal skills improvement with growth normalization. Schwantje et al. began treatment at 3 years in their patient and showed a stabilization of symptoms with improvement in growth curves, resolution of diarrhea but with persistence of motor problems. Montomoli et al. began supplementation before 3 years in one of their patient and demonstrated a stabilization of symptoms. In their other patients where treatment could not be instaured, patients died at 1 and 3 years old respectively.

All patients from our 3 families harbored the same intronic variation in the *SLC5A6* gene localized outside from canonical splicing sites. This variation is absent from large genomic database such as gnomAD (gnomAD v4) or deCAF. The 4 patients are not from the same family (except for patients 1 and 2) but all originated from the same world region (Algeria, Maghreb). This suggests a founder effect for this mutation in Maghreb. Thanks to RNA analysis of *SLC5A6* transcripts, we could confirm the deleterious effect of this variation, leading to the retention of 18 nucleotides in frame leading to a probable 6 AA insertion confirming the diagnosis of SMVT deficiency. The incorporation of 6 AA in frame within the fourth transmembrane domain of the transporter could led to destabilization of the protein or a mis-localization as previously described for some missense variations. Other pathogenic variations of the *SLC5A6* leading to SMVT deficiency have been proved to cause protein destabilization like *p*.Arg400Thr (first described by Byrne *et al* and then by Holling *et al*) or mislocalization like *p*.Arg123Leu (Subramanian *et al*). Byrne *et al.* showed a reduced biotin uptake due to *p*.Arg400Thr and Utsuno *et al* proved the protein instability due to *p*.Arg400Thr (located in the cytoplasmic facing region of transmembrane domain 10) based on measurement of free energy changes with FodlX. Subramanian *et al.* showed abnormal cellular localization for *p*.Arg123Leu (located in an extracellular loop between transmembrane domain 3 and 4). The fourth transmembrane domain is highly conserved between species and insertion of this putative 6 AA motif FFHMQQ is predicted to be deleterious by PROVEAN algorithm with a score of −12.5 (PROVEAN Protein (jcvi.org)).

Our study tends to prove that our patients present a complex neurophysiological phenotype with reduced sensory amplitudes although not fully abolished regarding the marked reduced motor amplitudes in lower limbs. However, if we only refer to the conduction studies and the foot deformities, this neuropathy could look like Charcot-Marie-Tooth disease. However, the disability progression and the additional clinical signs pointed to a complex neuropathy (Rossor et al., 2017). The delay to reach an accurate diagnosis and introduction of appropriate vitamin supplementation, as well as the short duration of treatment in patient 4 to date, prevented us to draw any firm conclusion regarding the effect of supplementation on disease course for the moment. 3D investigations on the variation effect and *in vitro* studies in cellular models have to be performed. Collaborations about those points are about to be performed.

## Data Availability

The original contributions presented in the study are included in the article/Supplementary Material, further inquiries can be directed to the corresponding author.

## Abbreviations

AA, amino acid; ACMG, American college of medical genetics; AAC, amino acids chromatography; ACPA, micro array DNA; cDNA, coding desoxyribonucleotide acid; CIPO, Chronic intestinal pseudo-obstruction; CMT, Charcot-Marie-Tooth; COMNB, childhood-onset biotin-responsive peripheral motor neuropathy; CSF, Cerebrospinal fluid; DNA, desoxyribonucleotic acid; mRNA, mature ribonucleic acid; MNGIE, Mitochondrial neurogastrointestinal encephalomyopathy; MRI, magnetic resonance imaging; MRSI, Magnetic resonance spectroscopic imaging; Mt DNA, mitochondrial DNA; NCS/EMG, (nerve conduction study and electromyography); NGS, next-generation sequencing; OA, optic atrophy; OAC, organic acids chromatography; PDH, pyruvate dehydrogenase; RNA, ribonucleic acid; SMVT, sodium dependent multivitamin transporter; SMVTD, sodium dependent multivitamin transporter deficiency; SRDDBC, spontaneously remitting developmental delay with brain cysts; VLCFA, very-long-chain fatty acids; WES, whole exome sequencing; WM, white matter; Y, years.

## References

[B1] Abrams A. J. Hufnagel R. B. Rebelo A. Zanna C. Patel N. Gonzalez M. A. (2015). Mutations in SLC25A46, encoding a UGO1-like protein, cause an optic atrophy spectrum disorder. Nat. Genet. 47, 926–932. 10.1038/ng.3354 26168012 PMC4520737

[B2] Byrne A. B. Arts P. Polyak S. W. Feng J. Schreiber A. W. Kassahn K. S. (2019). Identification and targeted management of a neurodegenerative disorder caused by biallelic mutations in SLC5A6. Npj Genomic Med. 4, 28–8. 10.1038/s41525-019-0103-x PMC6856110 31754459

[B3] Hauth I. Waterham H. R. Wanders R. J. A. van der Crabben S. N. van Karnebeek C. D. M. (2022). A mild case of sodium-dependent multivitamin transporter (SMVT) deficiency illustrating the importance of treatment response in variant classification. Cold Spring Harb. Mol. Case Stud. 8, a006185. 10.1101/mcs.a006185 35217562 PMC8958925

[B4] Holling T. Nampoothiri S. Tarhan B. Schneeberger P. E. Vinayan K. P. Yesodharan D. (2022). Novel biallelic variants expand the SLC5A6-related phenotypic spectrum. Eur. J. Hum. Genet. EJHG 30, 439–449. 10.1038/s41431-021-01033-2 35013551 PMC8747999

[B5] Horga A. Bugiardini E. Manole A. Bremner F. Jaunmuktane Z. Dankwa L. (2019). Autosomal dominant optic atrophy and cataract “plus” phenotype including axonal neuropathy. Neurol. Genet. 5, e322. 10.1212/NXG.0000000000000322 31119193 PMC6501639

[B6] Montomoli M. Vetro A. Tubili F. Donati M. A. Daniotti M. Pochiero F. (2023). A novel SLC5A6 homozygous variant in a family with multivitamin-dependent neurometabolic disorder: phenotype expansion and long-term follow-up. Eur. J. Med. Genet. 66, 104808. 10.1016/j.ejmg.2023.104808 37391029

[B7] Neophytou C. Pitsouli C. (2022). Biotin controls intestinal stem cell mitosis and host-microbiome interactions. Cell Rep. 38, 110505. 10.1016/j.celrep.2022.110505 35263602

[B8] Quick M. Shi L. (2015). “Chapter three - the sodium/multivitamin transporter: a multipotent system with therapeutic implications,” in Vitamins & hormones, hormones and transport Systems. Editor Litwack G. (Cambridge, Massachusetts, United States: Academic Press), 63–100. 10.1016/bs.vh.2014.12.003 PMC5530880 25817866

[B9] Richards S. Aziz N. Bale S. Bick D. Das S. Gastier-Foster J. (2015). Standards and guidelines for the interpretation of sequence variants: a joint consensus recommendation of the American college of medical genetics and genomics and the association for molecular pathology. Genet. Med. 17, 405–424. 10.1038/gim.2015.30 25741868 PMC4544753

[B10] Rossor A. M. Carr A. S. Devine H. Chandrashekar H. Pelayo-Negro A. L. Pareyson D. (2017). Peripheral neuropathy in complex inherited diseases: an approach to diagnosis. J. Neurol. Neurosurg. Psychiatry 88, 846–863. 10.1136/jnnp-2016-313960 28794150

[B11] Rupasinghe K. Onyeador N. (2023). Sodium-dependent multivitamin transporter defects: a rare cause of recurrent vomiting and faltering growth. Frontline Gastroenterol. 14, 346–349. 10.1136/flgastro-2022-102344 37409333 PMC11138166

[B12] Sabui S. Kapadia R. Ghosal A. Schneider M. Lambrecht N. W. G. Said H. M. (2018). Biotin and pantothenic acid oversupplementation to conditional SLC5A6 KO mice prevents the development of intestinal mucosal abnormalities and growth defects. Am. J. Physiol. Cell Physiol. 315, C73–C79. 10.1152/ajpcell.00319.2017 29669219 PMC6087731

[B13] Schwantje M. de Sain‐van der Velden M. Jans J. van Gassen K. Dorrepaal C. Koop K. (2019). Genetic defect of the sodium‐dependent multivitamin transporter: a treatable disease, mimicking biotinidase deficiency. JIMD Rep. 48, 11–14. 10.1002/jmd2.12040 31392107 PMC6606985

[B14] Subramanian V. S. Constantinescu A. R. Benke P. J. Said H. M. (2017). Mutations in SLC5A6 associated with brain, immune, bone, and intestinal dysfunction in a young child. Hum. Genet. 136, 253–261. 10.1007/s00439-016-1751-x 27904971 PMC5263180

[B15] Utsuno Y. Hamada K. Hamanaka K. Miyoshi K. Tsuchimoto K. Sunada S. (2023). Novel missense variants cause intermediate phenotypes in the phenotypic spectrum of SLC5A6-related disorders. J. Hum. Genet., 1–9. 10.1038/s10038-023-01206-5 38012394

[B16] Vadlapudi A. D. Vadlapatla R. K. Mitra A. K. (2012). Sodium dependent multivitamin transporter (SMVT): a potential target for drug delivery. Curr. Drug Targets 13, 994–1003. 10.2174/138945012800675650 22420308 PMC4406285

