# Supplementary material for: Recurrent “outsider” intronic variation in the SLC5A6 gene causes severe mixed axonal and demyelinating neuropathy, cyclic vomiting and optic atrophy in 3 families from Maghreb
Source: Front Genet. 2024 Jan 29;15:1352006. doi: 10.3389/fgene.2024.1352006 (PMC10859498; doi:10.3389/fgene.2024.1352006)
Supplement: Supplementary file 1 [file DataSheet1.pdf]

Supplementary material and method

Consent form for genetic analysis

Informed consent was obtained from each patient included in this study.

For patient 1 and 2

Patient Recruitment and Genetic Analyses.

Patients 1 and 2 were brothers. The parents gave written informed consent for the genetic study Whole exome sequencing (WES) was performed on genomic DNA from patients and parents on Illumina sequencing platform (NovaSeq6000) using the DNA prep with enrichment Illumina kit at Lille Hospital. Sequence reads were aligned to the human genome (hg38 assembly). Base calling was made using DRAGEN software. Variants were then annotated with an in-house pipeline (ANATOLE2) and AnnotSV for structural variants (Geoffroy et al., 2018). Splice variation were evaluated using Splice AI and SPiP tools. Coverage for these patients was 95.9% and 94.6% at a 20x depth threshold. Identified clinically relevant genetic variants were confirmed by Sanger sequencing.

For patient 3

The parents gave written informed consent for the genetic study.

Library preparation. Fragmentation of 100 ng of genomic DNA was performed using enzymatic fragmentation using Kapa library hyperprep kit following manufacturer recommendations (KAPABIOSYSTEMS, ROCHE). Then, enrichment for exonic sequences was performed using Hyperexome V.1 kit following manufacturer recommendations (NimbleGen, Madison, Wisconsin).

Sequencing. The captured libraries were sequenced on a Nextseq500 instrument (Illumina, San Diego, California, USA) with high-output FlowCell and reagent, in order to obtain 150 bp paired-end reads.

Bioinformatic analyses. Demultiplexing and .fastq files generation were performed using Bcl2fastq software (v.1.8). Alignment and variant calling were performed through an in-house pipeline following the BWA/GATK gold standard best practices. The variant calling files (VCF) were annotated using both Annovar and Clinical insight (Qiagen, Hilden, Germany) on hg19 reference genome

assembly and the interpretative analysis was performed on de novo or homozygous inheritance of rare variants. Alamut (Interactive biosoftware, Rouen, France) and IGV (Broad institute, Cambridge, MA 02142, USA) were used as viewers of aligned sequences. Mean depth of coverage was 96X for the proband, and 108X and 98X for the mother and the father. *In silico* pathogenic prediction was performed with MaxEntScan (http://genes.mit.edu/burgelab/maxent/Xmaxentscan_scoreseq.html) and Human Splicing Finder3.0 (http://www.umd.be/HSF/) for splicing effect predictions and SPLICE AI (SpliceAI Lookup (broadinstitute.org). Protein modification insertion was evaluated by PROVEAN algorithm (PROVEAN Protein (jcvi.org)).

Open source genomic databases. The public variation database gnomAD (Broad institute, Cambridge, MA 02142, USA, http://gnomad.broadinstitute.org, version 1 checked on version 4 also) was checked for presence and allelic frequency of the variation identified. We filtered variants according to their frequency in gnomAD. For recessive disorders we only kept variants under 5% of minor allele frequency (MAF) and for autosomic dominant ones those under 1% of MAF.

This variant has been registered in the Clinvar database VCV002507024.2 (VCV002507024.2 - ClinVar - NCBI (nih.gov)).

DNA extraction: on blood EDTA samples with whole blood cartridge Maxwell promega RSC according to manufacturer recommendations

Material and methods used for RNA study

Sanger sequencing. Sanger sequencing was used to sequence exon 4 to 6 on cDNA from SLC5A6 gene (NM_021095) after amplification of cDNA by PCR. Primers sequences and PCR conditions are available upon request.

RNA extraction and transcript analyses Total RNA was extracted from blood Paxgene tubes using Maxwell RSC extraction technology MAXWELL RSC SIMPLY RNA BLOOD KIT ref : AS1380 (Promega, Madison, Wisconsin, USA) following manufacturer recommendations or from fibroblasts culture of the proband, following manufacturer recommendations using MAXWELL RSC SIMPLY RNA TISSUE KIT ref : AS1340. cDNA was then generated and sequenced by Sanger sequencing.

cDNA synthesis: cDNA was generated from 400 ng of total RNA with 3.2 µL of water RNAase free, 2µL of RT Buffer 10X, 2µL of RT Random primers 10X, 0.8µL of dNTP mix, 1µL of Reverse transcriptase and 1µL of RNASe inhibitor incubated during 9min30sec, then 37°C during 120 min, and 5 min at 85°C and finally at 4°C storage (HIGH CAPACITY CDNA REVERSE TRANSCRIPTION KIT NO RNASE INHIBITOR ref : 4368814 - life technologies and HIGH CAPACITY CDNA REVERSE TRANSCRIPTION KIT NO RNASE INHIBITOR ref : 4368814 - life technologies)

Sanger sequencing.

Sanger sequencing was used to sequence cDNA generated in the patient 3 and her parents. Amplification through PCR was performed with primers targeted a fragment from exon 3 to 6 of the SLC5A6 gene: Forward primer exon 3: 5' CTGCATCTCACCAGTGCCTA and Reverse primer Exon 6: 5' ATAGACGGTACAGACAATGCCC

Transcript reference used for this study analysis is NM_021095.4 (MANE Select Canonical, ENST00000310574.8)

RT-Q-PCR: Quantitative PCR was performed on cDNA generated from RNA extracted from patient 3 fibroblasts and from reference samples fibroblasts from Cochin Cells Bank using SYBR green mix and amplifying targeted gene *SLC5A6* (same primers as those used for PCR and Sanger on cDNA) and on reference gene *ABL f*or the patient and for reference samples. Analysis is done thanks to Roche LC 480 software LightCycler® 480 SW 1.5.1 with method: Advanced Relative Quantification for ABL/SLC5A6. Each PCR is analyzed in triplicate.

For Patient 4

WES was performed as trio. Informed consent was obtained from each patient included in this study. For whole-exome sequencing, libraries were prepared from genomic DNA (3 µg) of parents and affected child using an optimised SureSelect Human Exome kit (Agilent). Captured, purified and clonally amplified libraries targeting exonic sequences were sequenced on a HiSeq 2500 (Illumina). Sequence reads were aligned to the human genome (hg19) using BWA software. Downstream processing was carried out with the Genome analysis (GATK), SAM and Picard Toolkits. Single

nucleotide variants and indels were subsequently called by the SAMtools suite (mpileup, bcftools, vcfutil). All calls with a read coverage ≤5× and a Phred-scaled single nucleotide polymorphism (SNP) quality ≤20 were filtered out. Substitution and variation calls were made with the SAMtools pipe- line (mpileup). Variants were annotated with an in-house Paris Descartes University bioinformatics platform pipeline based on the Ensembl database (release 67). De novo, recessive and X-linked inheritance was applied for candidate gene identification. After exome analysis, each selected variant was confirmed by direct sequencing using BigDyedideoxy terminator chemistry on an ABI3130xl genetic DNA analyzer (Applied Biosystems) after PCR. Primer sequences PCR conditions are available on request.
